# A New Tactile Transfer Cell Using Magnetorheological Materials for Robot-Assisted Minimally Invasive Surgery

**DOI:** 10.3390/s21093034

**Published:** 2021-04-26

**Authors:** Yu-Jin Park, Seung-Bok Choi

**Affiliations:** 1 Smart Structure and Systems Laboratory, Department of Mechanical Engineering, Inha University, Incheon 22212, Korea; eugene5059@inha.ac.kr; 2 Department of Mechanical Engineering, The State University of New York, Korea (SUNY Korea), Incheon 21985, Korea

**Keywords:** magnetorheological (MR) materials, tactile transfer cell, repulsive force, porous structure, human tissue, stress relaxation, viscoelastic property, viscoelastic model, psychophysical test

## Abstract

This paper proposes a new type of tactile transfer cell which can be effectively applied to robot-assisted minimally invasive surgery (RMIS). The proposed tactile device is manufactured from two smart materials, a magnetorheological fluid (MRF) and a magnetorheological elastomer (MRE), whose viscoelastic properties are controllable by an external magnetic field. Thus, it can produce field-dependent repulsive forces which are equivalent to several human organs (or tissues) such as a heart. As a first step, an appropriate tactile sample is made using both MRF and MRE associated with porous foam. Then, the microstructures of these materials taken from Scanning Electron Microscope (SEM) images are presented, showing the particle distribution with and without the magnetic field. Subsequently, the field-dependent repulsive force of the sample, which is equivalent to the stress relaxation property of viscoelastic materials, are measured at several compressive deformation depths. Then, the measured values are compared with the calculated values obtained from Young’s modulus of human tissue data via the finite element method. It is identified from this comparison that the proposed tactile transfer cell can mimic the repulsive force (or hardness) of several human organs. This directly indicates that the proposed MR materials-based tactile transfer cell (MRTTC in short) can be effectively applied to RMIS in which the surgeon can feel the strength or softness of the human organ by just changing the magnetic field intensity. In this work, to reflect a more practical feasibility, a psychophysical test is also carried out using 20 volunteers, and the results are analyzed, presenting the standard deviation.

## 1. Introduction

In this era of increasing interest in virtual reality, there have been attempts to implemented humans’ five senses as artificial media in various ways. In general, a human’s tactile sense can distinguish fine differences in texture, temperature, thinness and thickness, regardless of whether the materials are hard or soft. As such, the external sources that the human sense of touch can accept are very diverse, including vibration, heat and electricity; thus it is not easy to make a tactile sensation delivery device that adapts and reacts in various situations due to the wide range of human tactile sensations, so it is necessary to develop a very effective tactile transfer device. So far, tactile devices have been studied a lot as a means of communication for the visually impaired [1,2,3]. By expanding this point, the use of tactile information has been developed to a point where it is applicable in various industries [4,5,6,7,8]. In addition, a method of implementing tactile feedback using vibration mechanisms was studied [6,7] in which the tactile information can detect one or more properties such as softness, texture, shape, composition, shear force and normal force [9]. In the medical field, a study was conducted to provide tactile information to the surgeon by applying a device that realizes tactile sensation for robot surgery and laparoscopy surgery in the minimally invasive surgery field [10,11,12,13,14,15]. With the introduction of a laparoscope, an endoscope can be inserted to examine and treat the inside of the abdominal cavity. A small hole is made on the side of the abdomen, gas is injected to inflate it, and then a thin laparoscope is inserted. Since human organs are overlapped, removing the organs takes a long time and imposes a heavy burden on the surgeons. Therefore, robot-assisted minimally invasive surgery, which significantly reduces the burden on doctors and patients, is becoming more popular [16,17,18]. 

Robot surgery is used for surgery that requires elaborate work to minimize physiological damage and reduce pain to speed up recovery. However, robot-assisted minimally invasive surgery (RMIS) has disadvantages. When performing RMIS, the surgeon relies only on visual information to judge the progress of the operation. This is the biggest problem, surgeons argue. It is true that the view is partially concentrated and can be seen in detail, but it is inevitably narrowed, and the amount of information transmitted is inevitably limited [19,20]. That is why caution and care are needed, as the status must be judged by the visual information. Recently, to compensate for this, a technology that provides 3D imaging in real-time has been developed [20]. It tries to overcome the limitations of visual information transmission by increasing the quality of information using multiple cameras from various angles. However, it is certainly true that this approach is still limited to visual information. In general, a surgeon performing surgery can directly touch the area so that the exact condition of the surgical site can be immediately known during the operation. In contrast, in RMIS, the patient is surrounded by numerous machines. The surgeon is the master of the robot. However, a certain distance between the patient and the surgeon is inevitable. The inability to touch while the visual field is narrowed maybe the cause impairing quick judgment in urgent situations during surgery. Therefore, when RMIS is in progress, if tactile sensation information can be delivered to the surgeon, surgeons’ problems will be resolved to some extent. Therefore, it has been proposed that a haptic feedback system can be additionally mounted on the master part of the surgical robot or the controller so that data on the tactile feel of the internal organs can be obtained in real-time [11,21,22,23,24]. The haptic system is an important element that can increase the surgeon’s concentration during surgery. A lot of research is being conducted as the real-time information of the patient is transmitted. The inner tissues of the human body have varying degrees of rigidity and can change in real-time. Therefore, it should be possible to collect information on various organs and tissues’ tactile sensations with a single tactile feedback device. Considering these points, different tactile transfer devices have been studied [25,26,27,28]. The most widely studied is the use of shape memory alloy (SMA) wire technology [25,26]. However, since this method has a small stroke, if a mechanical amplification system is used to increase the required stroke, there may be a performance degradation limit. The second widely studied method is to use piezoelectric technology [27,28]. This method is usually produced as an array type. This method can present a wide range of forces and sufficient force, but the relaxation force is not measurable. The human body has viscoelastic properties, which are important for tactile information.

Moreover, if it conveys the human body’s internal state, it is not suitable because it cannot simply convey the degree of rigidity. Despite the high interest and research on various devices, they are not suitable for surgical robots or laparoscopy because of their lack of viscoelastic properties that transmit information about the patient’s surgical site. To compensate for these points, a pneumatic haptic feedback system was proposed [29,30,31]. The sense of touch was controlled using an actuator array and adjusting the air pressure in this task. Although it can express viscoelastic properties, it is inappropriate for expressing the properties of incompressible internal tissues due to air pressure compressibility. To overcome this problem, recently, a tactile device using a magnetorheological fluid (MRF) has been proposed for the possible application in RMIS [31,32,33,34,35,36]. MRF has several advantages, such as controllable viscoelastic properties due to the magnetic field, fast response time and low power consumption [37]. Therefore, MRF-based tactile devices can detect various human tissue organ characteristics by controlling the magnetic field strength. Moreover, the controllability can realize specific rigidity and damping characteristics with a simple open-loop control method.

**Figure 1:**
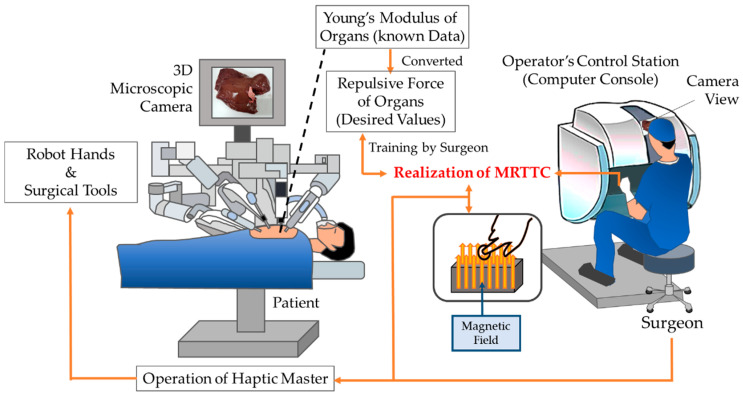
Conceptual diagram of the proposed MRTTC: how to transfer tactile information during robot-assisted minimally invasive surgery.

Figure 1 shows a schematic diagram of the proposed MR materials-based tactile transfer cell (MRTTC in short), which presents how to transfer tactile information between the surgeon and patient’s organs during RMIS. It is challenging to measure the repulsive force in real-time using force sensors during surgery. Thus, the repulsive force of each organ, which is generally known as Young’s modulus, is converted to the repulsive force of each organ. In this work, the finite element method is used for this conversion. Then, the surgeon needs to train for some time in order to recognize the repulsive force of each organ using the proposed MRTTC by referring to the desired values stored in the computer. Of course, the surgeon should know how to generate the desired values from MRTTC through the adjustment of the magnetic field intensity to be applied to MRTTC. Once the surgeon can adjust the magnetic field to achieve the desired value corresponding to a specific organ, the surgeon operates the haptic master based on both the microscopic camera view from the console and the repulsive force felt from MRTTC. 

It is noted here that the surgeon can quickly change the repulsive force during the surgery by changing the magnetic field and compressive deformation of MRTTC, if necessary. Recently, several studies have been conducted to simulate human organs and tissues using MRF. MRF sponge was studied to mimic human organs and tissues [38], and MRF absorbed in polyurethane foam was proposed to investigate the reaction force characteristics of various organs and tissues [39]. The fabrication and modeling of the MRF sponge actuator were studied to mimic human tissue [40]. In addition, Young’s moduli of human organs were converted to the repulsive force and compared with the measured values from the MRF sponge. To compare the MR sponge’s repulsive force, human data was calculated by finite element analysis and compared with experimental values [41]. Despite several works on tactile devices utilizing MRF, there are some impediments to be overcome for practical use. In particular, the sealing problem to prevent the leakage of MRF from the porous foam is the most serious one in MRF-based tactile devices. Research was conducted to produce a new MRE type by confining MRF in a silicone resin [42] and the study applied to the isolator type as a structure in which MRF is confined in the silicone was also studied [43]. Recently, research was carried out to increase the controllable range of the generated force by encapsulating MRF with MRE [44]. In addition, a tactile device using MRF as a core and the silicone rubber as a cover was proposed and its tactile performance was investigated experimentally. In this work, the repulsive force is generated from the particle formation of MRF only, but the repulsive force of the proposed MRTTC is generated from the particle chain-like formation of MRF core and the particle interaction of MRE cover simultaneously. In addition, the tactile shape of the previous work [41] is rectangular, but the shape of the proposed MRTTC is a hemispherical type which can provide better uniformity of the magnetic field distribution.

Consequently, the main technical contribution of this work is to propose a new type of MR material-based tactile transfer cell (MRTTC for short), which features easy fabrication, excellent sealing, manufacturing uniformity, a wide range of field-dependent repulsive forces and a short training time. The proposed MRTTC is capable of generating and transmitting various repulsive forces to the surgeon that occur in the surgical field of laparoscopic surgery or RMIS. To achieve this goal, a tactile sample is made by absorbing MRF into a porous polyurethane-type foam and then encapsulating it with MRE ensuring perfect sealing. Before preparing the sample, a uniform distribution of the magnetic field is carefully checked through the magnetic field analysis to select an appropriate shape of MRTTC. Subsequently, an experimental apparatus for testing the repulsive force of the sample, in which the stroke in the vertical direction is controllable, is established. In this test, the load cell and the laser sensor are used to measure the repulsive force and compressive depth, respectively. The magnitude of the repulsive force of MRTTC due to the magnetic field is measured as a function of time to observe the viscoelastic properties, which can characterize several human tissues as the property of stress relaxation. To obtain a wide spectrum of repulsive forces, MRTTC is tested with three different deformation depths, and the measured values are compared with the calculated values from the finite element method. In this calculation, Young’s modulus of human tissues given in the reference is converted to the corresponding repulsive force considering that the measured repulsive force is modeled by the stress relaxation behavior. Especially, the converted repulsive force is calculated as the maximum repulsive force compared with the highest value in the stress relaxation result. After validating the measured values of the repulsive forces considering the hardness of human organs, a psychophysical test is carried out to reflect the practical feasibility of the proposed MRTTC. 

## 2. Characteristics of Materials

### 2.1. Magnetorheological Fluid

MRF, the primary material of MRTTC, is a magnetorheological material containing fine iron powder in an oil base such as silicone. The properties of MRF vary according to the type of base oil and the weight fraction of iron particles. In general, a higher particle concentration provides a higher yield shear stress. The particles are randomly distributed in the absence of a magnetic field, but the particles form chains with the magnetic field. The stronger the magnetic field, the more rigid chains are formed. This salient property can be used to control the yield shear stress of MRF depending on the strength of the magnetic field. Besides, the type of MRF operation is largely divided into three modes: valve mode (flow mode), shear mode and squeeze mode [45]. In all modes, the magnetic field acts perpendicular to the plate plane, restricting the fluid inside the plate. Each mode can be applied according to the main operation of MRF in application systems or devices. For example, the flow mode (valve mode) is suitable for dampers and shock absorption because it controls the channel to which the magnetic field is applied. The shear mode is mainly applicable to rotating devices such as brakes and clutches.

**Figure 2:**
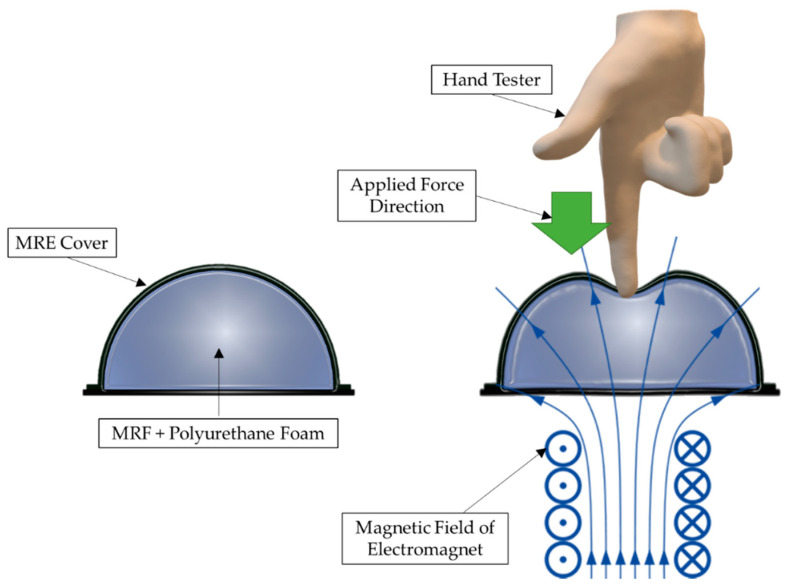
Squeeze mode of MRTTC.

**Figure 3:**
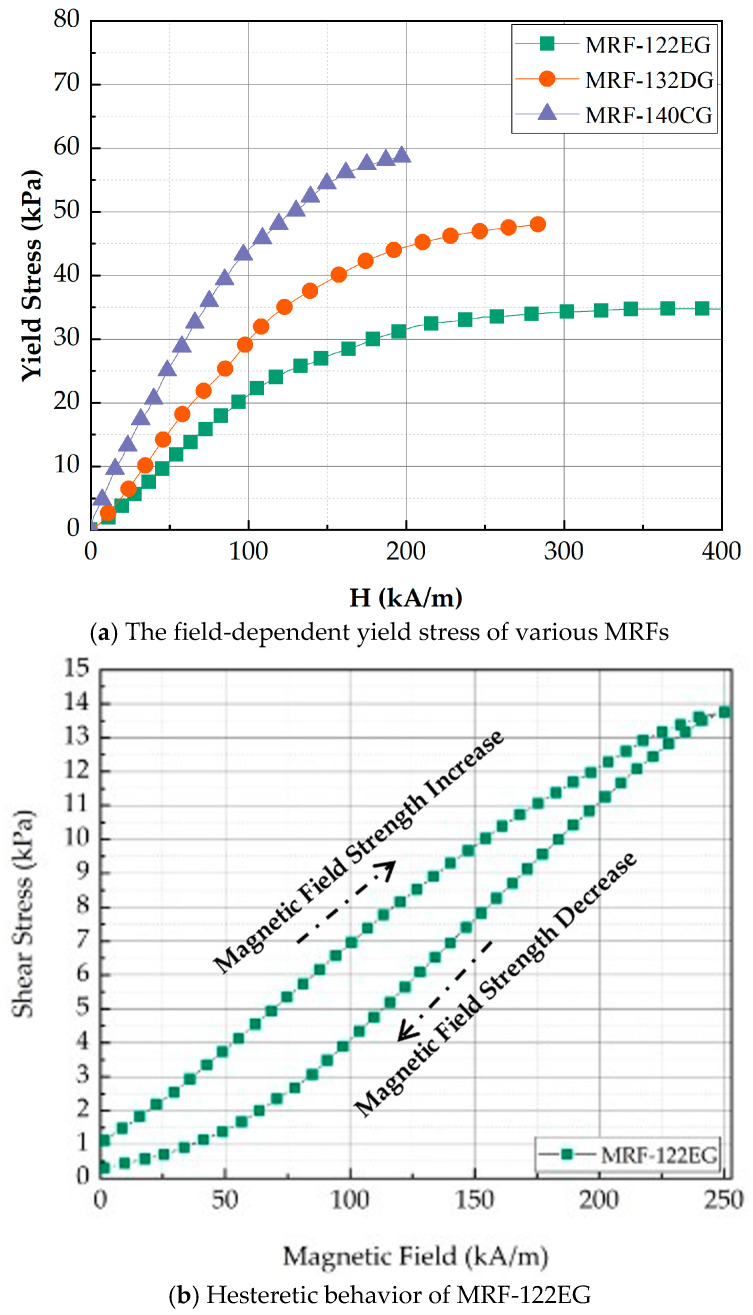
Properties of Magnetorheological Fluid.

The squeeze mode of MRTTC, shown in Figure 2, which represents the compressive operation, is applied in small amounts for vibration control and tactile devices for RMIS. Especially, the squeeze mode is most suitable for a tactile device because the hand exerts force in the same axis as the direction of the magnetic field, as shown in Figure 2. The repulsive force is generated according to the deformation of the MRTTC as a force is applied. As shown in the right figure in Figure 2, the hand exerts a force on the same axis where the magnetic field of the MRTTC is applied. When the squeeze mode is used as the axis of the same force in the direction of the magnetic field, the repulsive force can be variously reproduced with the rigidity of the chain generated vertically when the force is applied. When a constant force of constant displacement is applied, the relaxation stress can propagate through the chain rearrangement of particles in the MRTTC. Due to the rearrangement of the chain, it is possible to mimic the viscoelastic properties of the internal organs of the human body and to deliver more accurate tactile information. Therefore, this study utilizes the squeeze mode of MRF for the fabrication of MRTTC and undertakes an experiment on the possibility of reproducing the tactile sensation of human internal organs. In this work, among several types of MR fluids which are commercially available from Lord Corporation (Cary, NC, USA), MRF-122EG is chosen since it has been proven that this fluid has appropriate yield stress to mimic human internal tissues with a low current (magnetic field) [42]. Figure 3a shows the field-dependent yield stress of three different MR fluids manufactured by Lord Corporation. When comparing the yield stress with MRF-132DG and MRF-140CG, frequently used for dampers, MRF-122EG has lower yield stress. This is a positive property to mimic the soft tissue of human organs such as the kidney. In addition, the initial value is expected to be low due to the low density. Figure 3b shows the hysteretic behavior of the MRF-122EG due to the loading and unloading of the magnetic field. It is clearly seen that MRF exhibits viscoelastic behavior with respect to the external magnetic field. It is noted here that when MRF-122EG is used with the polyurethane foam as the frame of MRTTC, it produces a low viscosity to express the soft human body’s internal structure. MRF-122EG has a particle weight of 72%, density of 2.28–2.48 and the yield stress of 32 kPa at 200 kA/m. 

### 2.2. Polyurethane Foam (Porous Structure)

**Figure 4:**
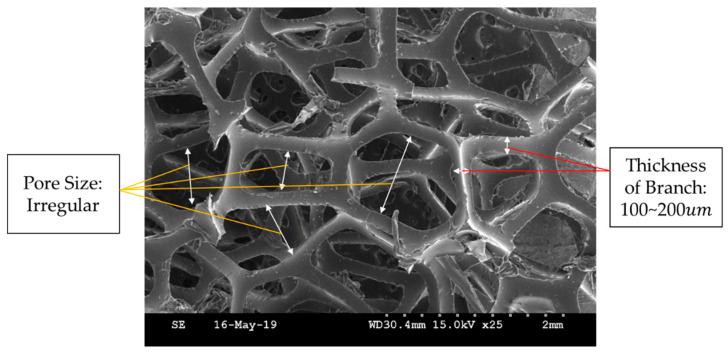
SEM photo of polyurethane foam.

Polyurethane foam is effective in maintaining the shape of the tactile device, and hence it has been used as a frame for MR tactile devices [39,40,41,42]. It consists of many dense pores of irregular size. These pores absorb and trap MRF. Because this foam is resilient, it can help the resilience of MRTTC when the external pressure is removed. Polyurethane foam is sealed, and MRF is injected to make MRTTC. The foam used in the manufacture is a polyurethane foam of 25 ppi (pore per inch). Figure 4 is a SEM image of 25 ppi polyurethane foam. It is clearly seen from Figure 4 that branches connect irregular pores. The pore size is uneven. Branches of about 100~200 um thick are entangled and constitute pores. MRF is absorbed inside these uneven pores. However, since it is an open-cell type, so there is a point to note about sealing. In order to prevent the leakage of MRF, a cuboid and hemispherical form of MRTTC is chosen in this work. The size, of course, is determined by considering the force control range according to the initial repulsive force and magnetic field. In addition, to apply a uniform magnetic field, the magnetic field analysis has been conducted, and the polyurethane foam of an appropriate shape is selected. Furthermore, when the external force is removed, the shape of the pores recovers over time, and hence it concentrates attention on the elasticity.

### 2.3. Magnetorheological Elastomer

When manufacturing the proposed MRTTC using polyurethane foam, the open type is used. Hence, there is a need to seal the absorbed MRF to the outside. There are two approaches to resolve this problem. One is to use an existing packaging material like low-density polyethylene (LDPE) [46], and the other approach is to use MRE, which consists of the iron particles in a base matrix such as silicone rubber. It is known that LDPE has excellent oil resistance, toughness, flexibility, and relative transparency and hence is frequently utilized in packaging for food and non-food purposes as a protective film [46]. However, there is a sealing problem. Leakage of MRF may occur between the tapes when sealing the polyurethane foam with LDPE. Furthermore, the shape restoration cannot be performed properly due to the permanent deformation of the surface when used for a long time. Therefore, in this work, MRE, which is recognized as a smart material, is chosen instead of LDPE. When the magnetic field is applied to MRE, interactions between magnetic particles are created. Carbonyl iron powder (CIP), which is unevenly distributed, interacts according to the direction of the magnetic field, and the stiffness is changed accordingly [47]. In other words, the stiffness of MRE can be controlled by the intensity of the magnetic field. Therefore, the use of MRE can bring both excellent sealings to prevent the leakage of MRF and a controllable parameter to adjust the repulsive force of the proposed MRTTC. The MRE to be fabricated in this work is thinner than LDPE, and hence it can be easily used for wrapping the polyurethane foam. As a matrix, silicone rubber is chosen as a primary material for making MRE since it is a good candidate to make a certain shape. Considering the hardness of various silicone rubbers, a flexible and stable material, which can simulate the human tissues, is chosen. An Ecoflex silicon rubber of suitable hardness (Ecoflex 00–30, Smooth-On, Inc., Macungie, PA, USA) is adopted in this work. It is noted that the silicone rubber chosen in this work is frequently used for human organ models and makeup because it has good elasticity and stability.

**Figure 5:**
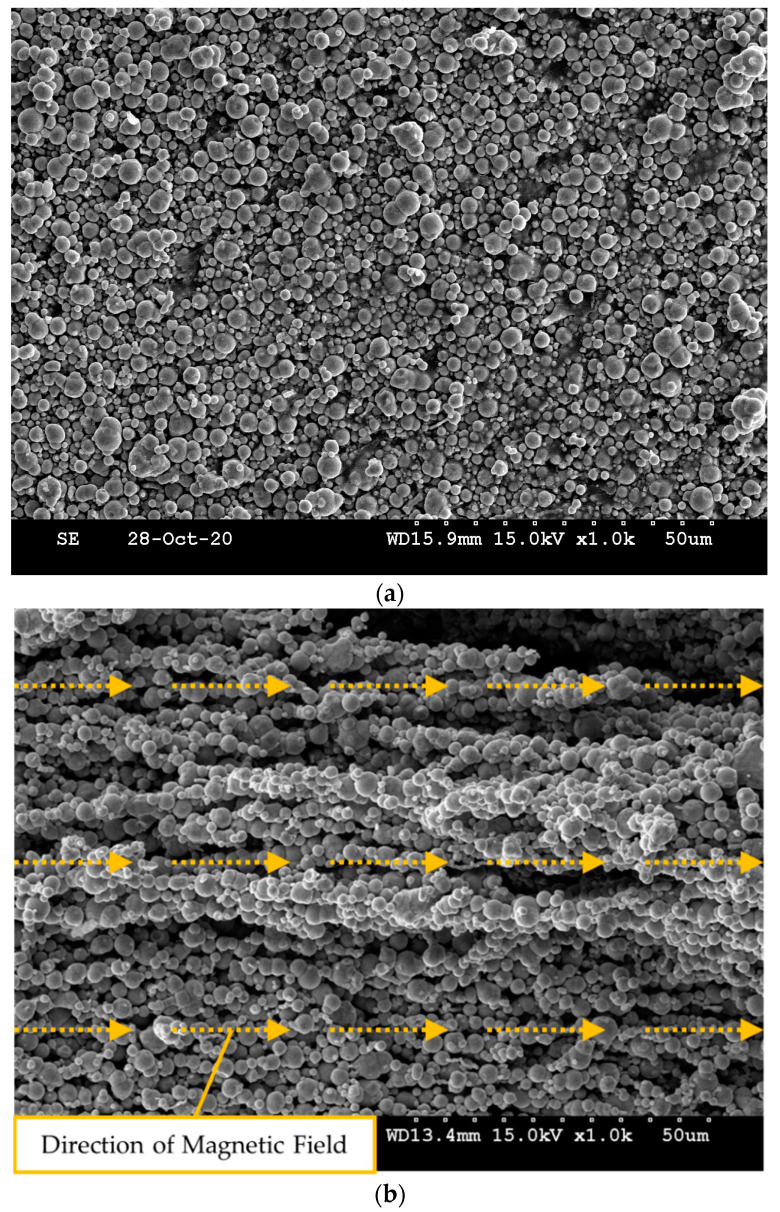
CIPs distribution of MRE (×1000): (**a**) magnetic field OFF, (**b**) magnetic field ON (0.2 T).

To make MRE, magnetic particles called carbonyl iron powder (CIP) are mixed with the adopted silicon base. In this work, CIPs of 1~5 um size made by BASF Corporation (Ludwigshafen, Land Rheinland-Pfalz, Germany) is used. Figure 5 shows SEM images of the particle distribution without and with the magnetic field. It is clearly observed that when the magnetic field is formed, the particles are arranged in a chain in the direction of the magnetic field. In fact, this material generates mutual attraction between the particles due to the magnetic field. Thus, the stiffness of MRE can be changed by the magnetic field intensity. In this work, MRE is manufactured with 50 wt% of CIP on the silicone base. This procedure can be summarized as follows: (a) The base material and the hardener are mixed in a 1:1 ratio. (b) Before blending, subdivide CIP by weight percentage and add it to the silicon. (c) When adding to the silicon using a skimmer, put the particles so that they do not clump together. (d) Blend for 30 min with a stick. (e) Prepare a mold to make the desired shape. (f) Pour the prepared MRE into the mold and remove the mold after 4 h of elapsed time. It is noted here that the mold is made by the 3D printer (3DP-310F, Cubicon, Gyeonggi-do, Korea) in this work. It is also remarked here that the mold is made to have 1 mm thickness using the acrylonitrile butadiene styrene copolymer.

**Figure 6:**
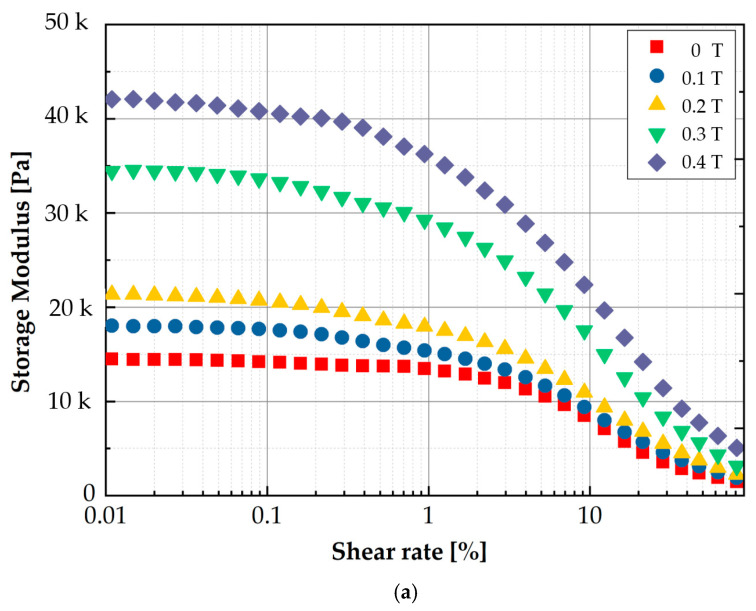

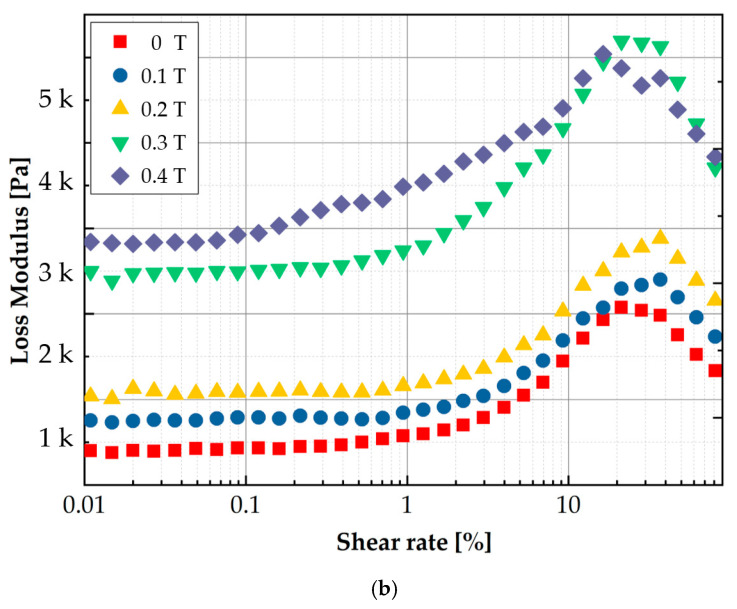
Measured viscoelastic properties of MRE (50 wt%): (**a**) storage modulus (**b**) loss modulus.

**Figure 7:**
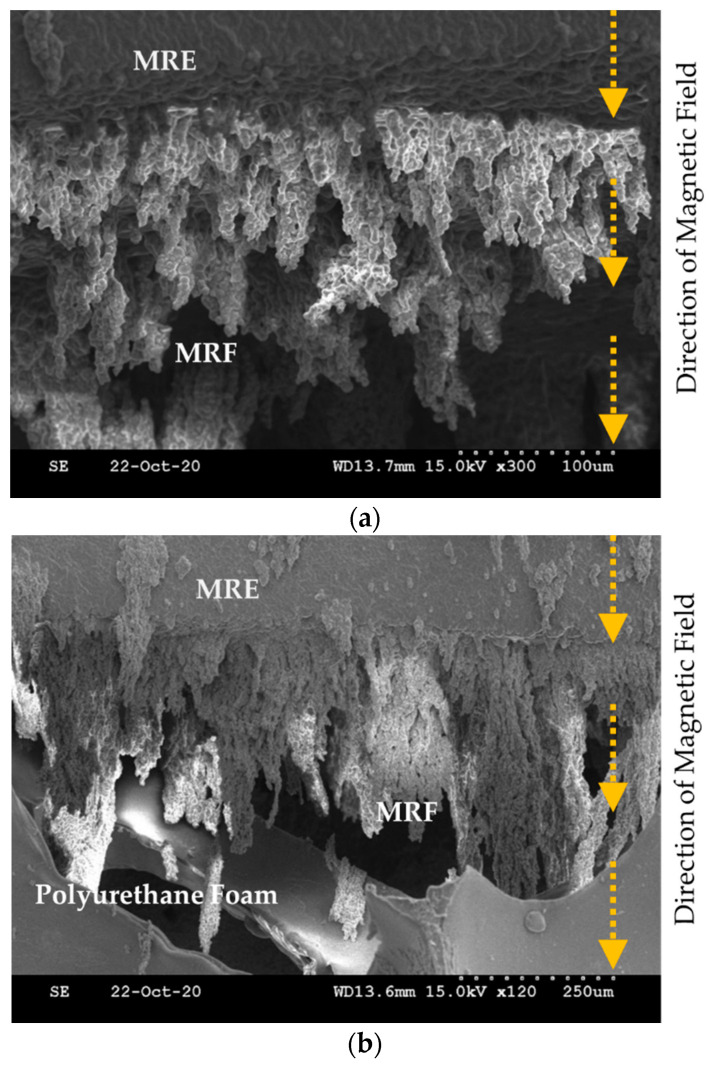

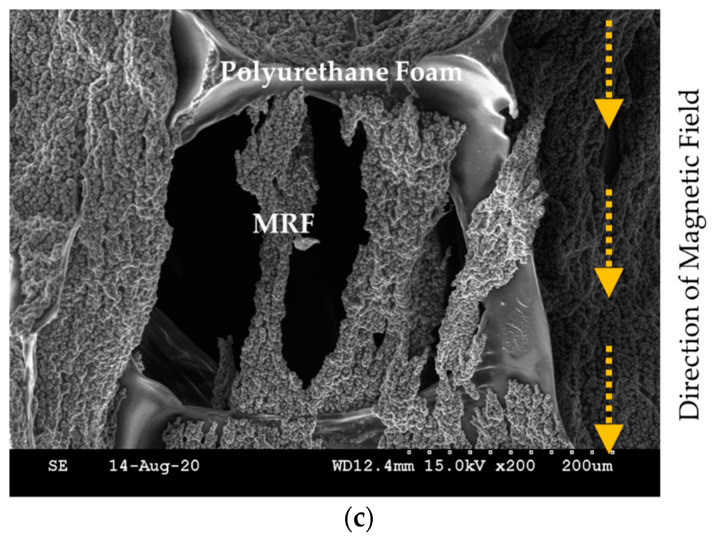
SEM photos of the particle distribution of MR materials under the magnetic field: (**a**) MRE + MRF, (**b**) MRE + MRF + polyurethane foam, (**c**) MRF + polyurethane foam.

To measure the MR effect of the manufactured MRE, the storage modulus and loss factor are measured using an MCR 302 rheometer (Anton Paar, Graz, Austria). Figure 6 presents the measured results for the modulus values. As well known, the storage modulus represents the elasticity, while the loss modulus represents the viscosity. The measured values show that both the storage and loss moduli are increased by increasing the magnetic field. This behavior directly indicates the MR effect of MRE like MRF. In other words, MRE exhibits the hysteresis behavior according to the loading and unloading of the magnetic field. As mentioned earlier, the proposed MRTTC consists of MRF, MRE and polyurethane foam. When the polyurethane foam is covered with MRE, MRE and MRF are in contact with each other. Thus, the material properties of both MRF and MRE are affected by the magnetic field. To visually investigate the interaction between MRE and MRF, SEM images are taken on the sample in the presence of the magnetic field. Figure 7 presents SEM images of three different components to which the magnetic field is applied in the vertical direction. Figure 7a shows that MRF particles form the chain structures on the MRE surface according to the magnetic field direction. Figure 7b shows how the chains are formed when the polyurethane foam is added to MRE and MRF. It is seen that MRF chains extended to MRE are formed by reaching the point of the polyurethane foam. Figure 7c presents how the chains of MRF particles in the pores are formed. MRF chains interact with CIPs of the matrix of MRE to form the chains, and CIPs of MRF inside the polyurethane foam pores cross the branch of the polyurethane foam to form the chain. Therefore, it is expected that a wide range of repulsive forces could be realized through the field-dependent particle interactions of the proposed MRTTC. It should be remarked that MRF in the porous structure is well contained without the leakage by MRE, whose base matrix is thin silicone rubber.

## 3. Fabrication of MRTTC

**Figure 8:**
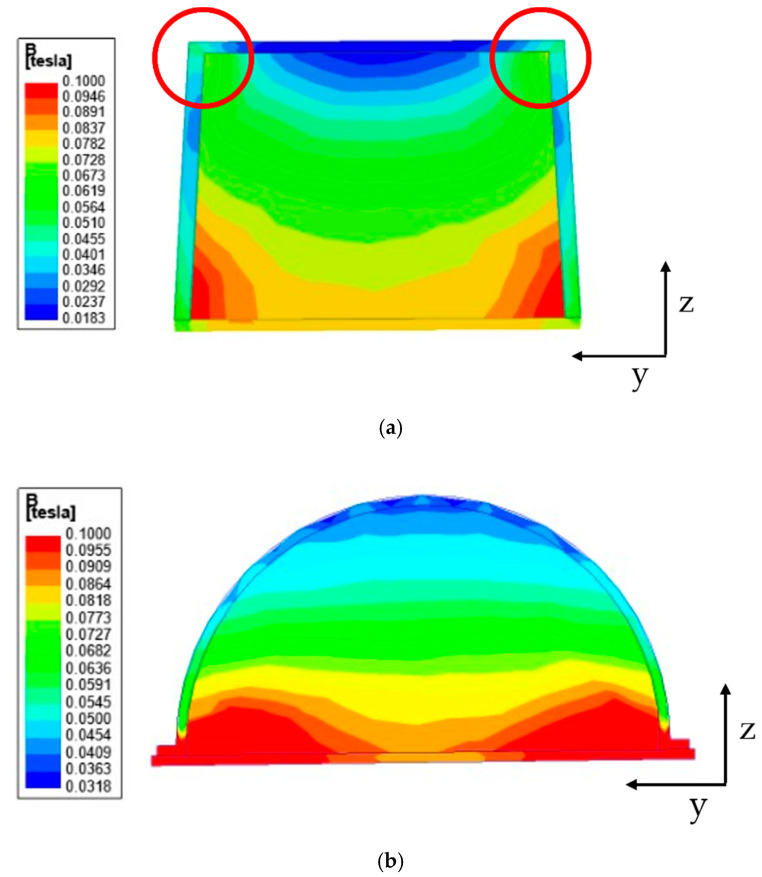
Magnetic field analysis results: (**a**) rectangular parallelepiped type, (**b**) hemispherical type.

In previous works, a cuboid-shaped MR sponge tactile device has been made [40,41] since its manufacture is relatively simple without the requirement of a mold. However, it has been observed that it is very difficult to recognize whether the magnetic field would be evenly distributed when the chains of MRF are formed. In this work, before deciding the device shape, the magnetic field distribution is carefully examined through a magnetic field analysis. Figure 8 shows the magnetic field analysis according to the device shape. In this work, the field-dependent repulsive force of MRTTC was measured in the vertical direction only and hence the magnetic field distribution is also carried out considering the vertical direction from top to downward. Figure 8 shows the magnetic field distribution of two different shapes: rectangular and hemispherical type. This analysis was undertaken using the finite element software of ANSYS Maxwell Program. Figure 8a is the analysis result of the rectangular parallelepiped. In the results, the blue color denotes the low intensity of the magnetic field. It is clearly observed from Figure 8a that the magnetic field at the circled edges (corners) is formed as an arch type resulting the high intensity of the magnetic field, while the field intensity at the position between two circles to be touched by fingers is very low compared to the edges. Therefore, it is hard to achieve sufficient repulsive force with the application of a low magnetic field (0.2 T) from this rectangular shape. Figure 8b presents the magnetic field distribution of the proposed hemispherical type. It is seen from the result that the magnetic field intensity is uniformly distributed with respect to the z-axis to be touchable by fingers. This result directly indicates that the proposed shape of MRTTC can produce higher repulsive force than the rectangular type under the same magnetic input and also more accurate force during the repeated operation due to the uniform distribution of the magnetic field intensity.

**Figure 9:**
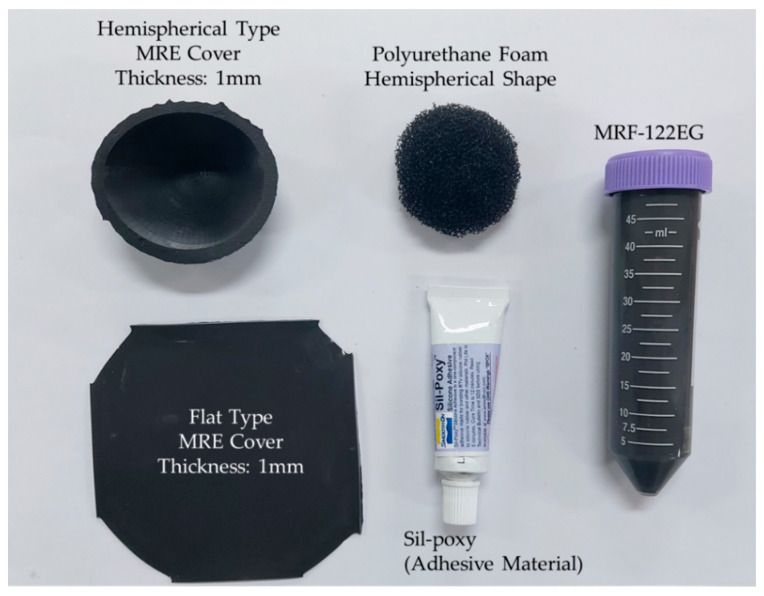
Components photos of the proposed MRTTC.

The repulsive force produced from the proposed MRTTC, which consists of MRF and MRE, is a function of the magnetic field. In other words, the repulsive force is produced from both the chain-like particle formation of MRF and particle interaction of MRE due to the magnetic field. The magnitude of the repulsive force depends on the magnetic field intensity. It is noted that the magnetic field intensity is relatively low (about 0.2 T) to form the particle chains and to activate particle interaction and hence it does not affect any other magnetic-responsive sensor or medical device during surgery. Based on this, the capacity and type of the electromagnet is determined. According to the electromagnet holder size, it is set in a hemispherical shape with a diameter of 50 mm and a height of 25 mm. This size, of course, depends on the core area and maximum magnetic field to be applied to MRF and MRE. As a result of the magnetic field analysis, it has been confirmed that the magnetic field lines are evenly distributed, unlike the rectangular shape, as shown in Figure 8. In this magnetic field analysis, 0.2 T is applied to both shapes. It is noted here that the electromagnet is the same type as that to be used for the measurement of the repulsive force of MRTTC. It is also remarked that the fabrication of the hemispherical shape is simple using an appropriate mold and conveniently to touch by the operator’s finger or palm. Figure 9 shows components for the complete fabrication of MRTTC. The open-type polyurethane foam is wrapped with MRE made with a thickness of 1 mm. The hemispherical polyurethane foam is embedded in the cover that can wrap the hemispherical shape. MRF-122EG is absorbed into the polyurethane foam and sealed with flat type MRE with the adhesive (Sil-poxy, Smooth-On Inc., Macungie, PA, USA).

## 4. Repulsive Force Calculation 

**Figure 10:**
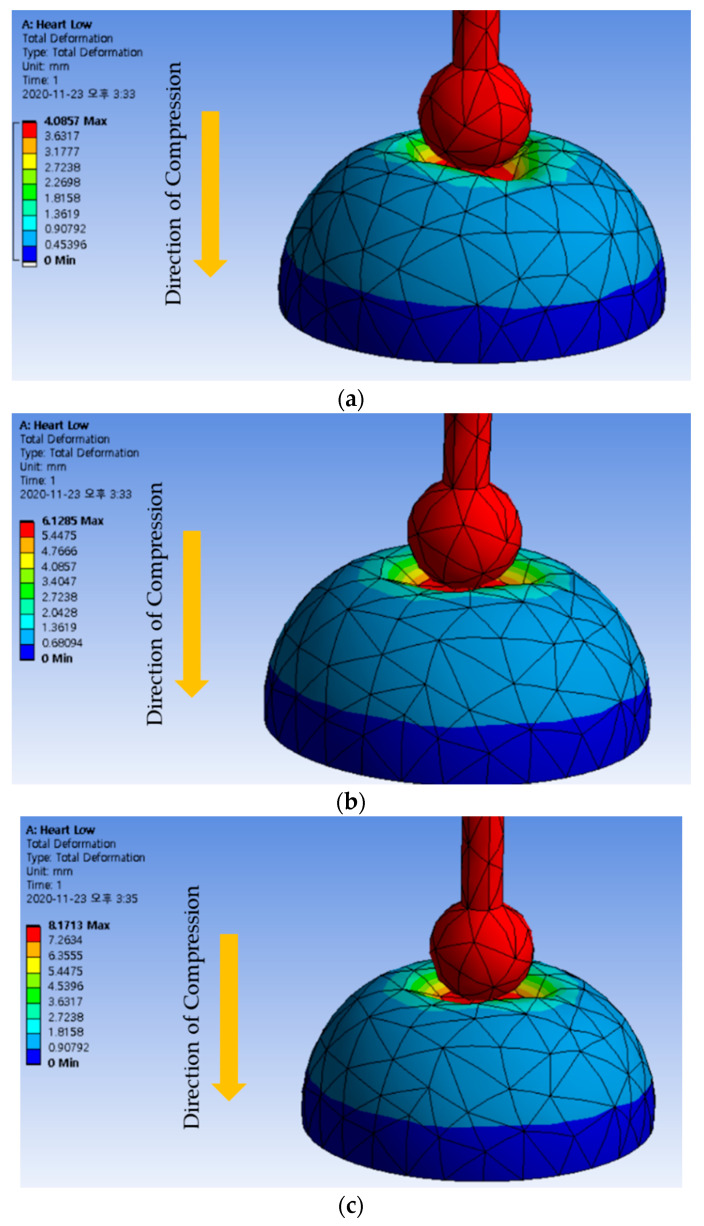
FEM analysis results according to compressive deformation depth (**a**) 4 mm compression. (**b**) 6 mm compression (**c**) 8 mm compression.

In order to validate the measured repulsive force produced from the proposed MRTTC, human reference data are required for comparison. Therefore, the finite element method (FEM) is utilized on the hemispherical geometry to convert Young’s moduli of human organs to the corresponding repulsive force. ANSYS Maxwell program is used for the analysis and the mesh is automatically generated: a total of 2196 elements are used. The following method is used to collect data on the control group. A 3D model made with the same shape and size of the produced MRTTC is used in FEM analysis. More specifically, the compression degree is set at 4, 6 and 8 mm, which is exactly the same as test conditions for the repulsive force measurement. Young’s modulus data for two types of muscles, kidney, skin and heart, given in [48,49,50], are used as shown in Table 1. The calculations from FEM are performed to find the maximum repulsive force during compression. The maximum repulsive force in the vertical (Z) direction is calculated using FEM software (Workbench, Ansys, Inc., Canonsburg, PA, USA). The maximum compression depth for FEM analysis is fixed by 4, 6 and 8 mm, as shown in Figure 10. The legend on the left is the displacement that has been transformed or moved from its original shape by pressing the device. Therefore, the area of the red color indicates the maximum deformation (or displacement) that occurred during the analysis. All results can confirm that an appropriate deformation is achieved from the hemispherical shape according to the compression depth. The calculated results for each compression depth are given in Table 2. The results in Table 2 indicate the maximum repulsive force for each compression depth. The spectrum of the calculated repulsive forces is to be compared with the spectrum of the measured values using MRTTC in a subsequent section to demonstrate the practical feasibility. 

## 5. Experimental Investigation

### 5.1. Experimental Set

**Figure 11:**
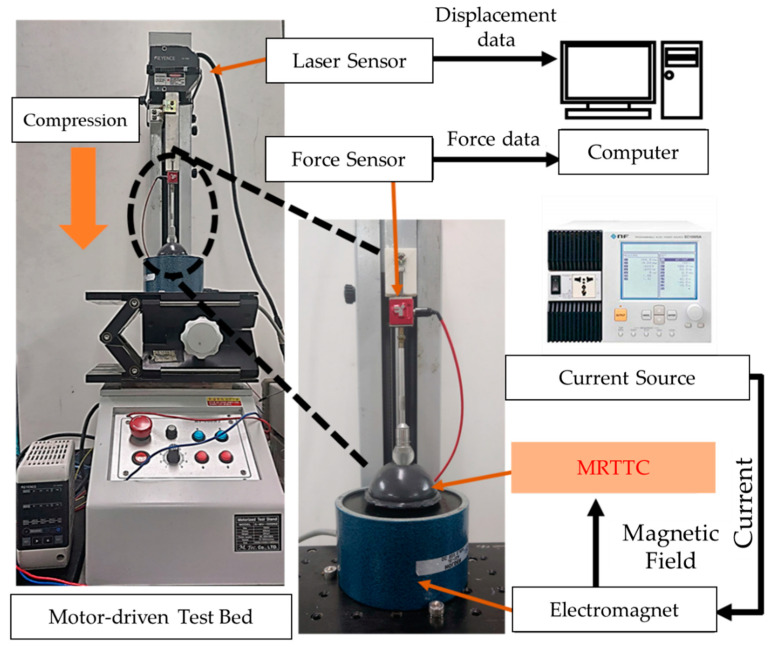
Experimental configuration for the repulsive force measurement of MRTTC.

To measure the field-dependent repulsive force of the proposed MRTTC, an experimental apparatus is established, as shown in Figure 11. The test stand is set at a constant speed of 0.4 mm/s (K-MV-1000N2, Measuring Technology Co. Ltd., Gyeonggi-do, Korea). The finger-acting end effector is connected to the force sensor. The final effector is manufactured in a spherical shape by covering the resin with aluminum, which is a non-magnetic material not affected by the magnetic field. The hemispherical end effector has a diameter of 15 mm. The force sensor used in this work is the type of load cell that can measure from 0.01 N up to 22.2 N (S-BEAM JR. Load Cell, FUTEK, Cor, Irvine, CA, USA). The vertical direction only is considered to measure the repulsive force since it is the dominant direction of the testing machine to match the calculated values using FEM shown in Figure 10. It is noted here that the fixture holding the force sensor and end effector is made of acrylonitrile butadiene styrene copolymer (ABS) using 3D printing technology. A laser sensor (LKG-30, Keyence, Cor, Osaka, Japan), which has a resolution of 0.001 mm, is used for the measurement of the accurate distance of compressive depth. The data measured by the laser sensor are collected according to the moving distance of the motor stand. The motor is set to be automatically stopped when the compressive depth reaches the set values of 4 mm, 6 mm and 8 mm. The speed of the end effector is set by 25 mm/s. Subsequently, the repulsive force is measured for 180 s and presented in the time domain. The compression time is maintained to obtain sufficient relaxation time. The electromagnet used in this work can produce a magnetic flux density of up to 0.2 T. (JL-10A, DC90, JL. Magnet Co., Seoul, Korea). The magnetic field generated should be uniformly formed in MRTTC. The magnetic field strength to be applied to MRTTC is chosen from 0 to 0.4 A in 0.13 A interval. Converting the value of each current to Tesla yields the following: 0 A ⇔ 0 T, 0.13 A ⇔ 0.065 T, 0.26 A ⇔ 0.13 T, 0.4 A ⇔ 0.2 T 

The force sensor installed on the moving piston, as shown in Figure 11 is used to measure the field-dependent repulsive force in the vertical direction at different compressive deformation depths. Once we have trained with the proposed MRTTC for feeling a certain repulsive force at the specific current, we can expect a certain repulsive force from MRTTC subjected to unknown input currents. To validate this, a psychophysical test has been carried out in this work. In short, one can predict a certain repulsive force at a specific input current after training and the force can be compared with the one measured from the force sensor.

### 5.2. Results and Discussion

**Figure 12:**
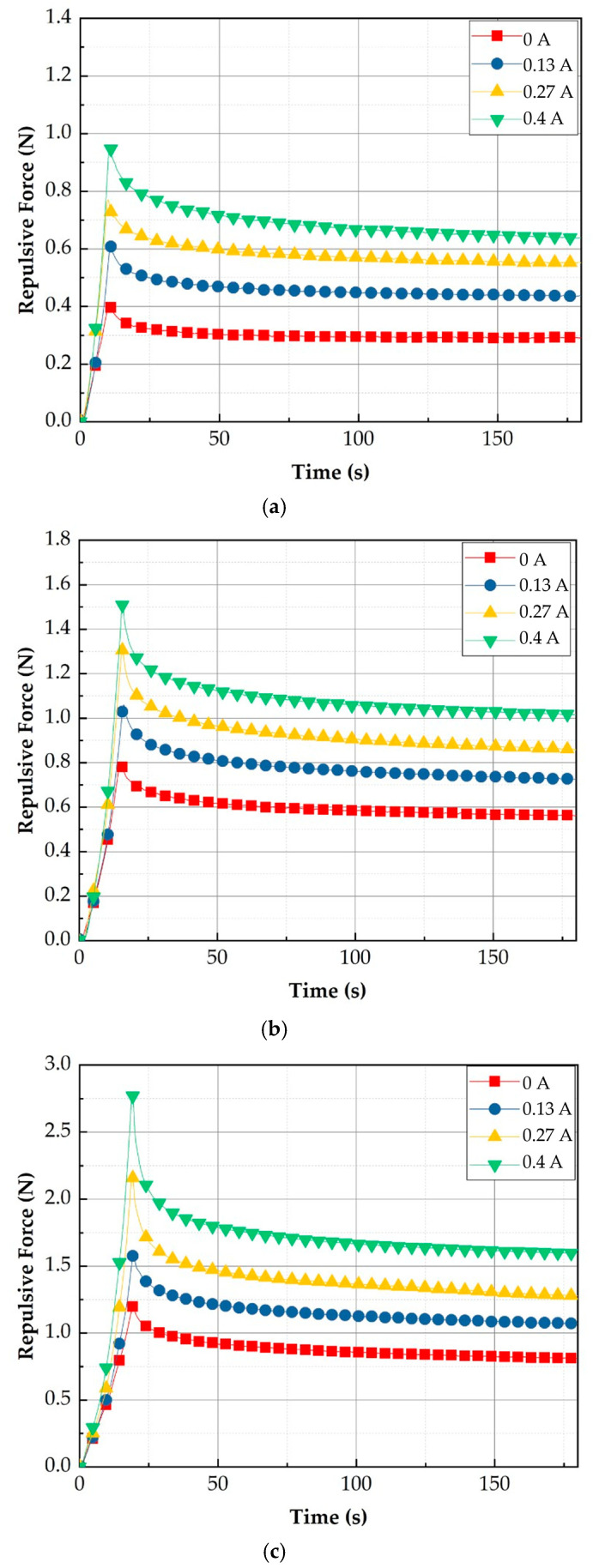
Measured repulsive forces at different compressive deformation depths: (**a**) 4 mm compression, (**b**) 6 mm compression, (**c**) 8 mm compression.

**Figure 13:**
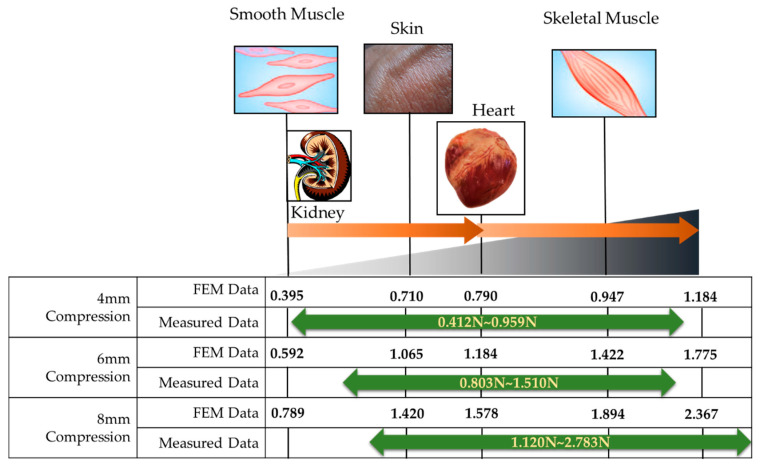
Comparative schematic of human tissues between calculation and measurement.

Figure 12 shows the measured repulsive forces at different compression depths. It is clearly observed that the repulsive force of each case increases as the input current (or magnetic field intensity) increases. The detailed specifications of the repulsive force are summarized in Table 3. The spectrum of each experimental result is identified as 0.412~0.959 N for 4 mm data, 0.803~1.510 N for 6 mm data, and 1.120~2.782 N for 8 mm data, depending on the peak value. The relaxation values are identified by 0.290~0.626 N for 4 mm data, 0.554~0.997 N for 6 mm data, and 0.791~1.567 N for 8 mm data. It is also seen that the transient peak value exponentially decreases before converging to the constant state value. This result directly indicates the physical behavior of human tissues (organs), which is called the stress relation phenomenon. In order to demonstrate the practical feasibility of the measured values, a comparative diagram between the measured and calculated values is drawn indicating several human organs and shown in Figure 13. It is confirmed from the comparison that the combination of the depth 4 mm with the depth 8 mm can provide a wide spectrum of the repulsive force covering most human organs. However, the proposed MRTTC needs to be modified a little to entirely capture the low repulsive force of soft tissues such as smooth muscles. This can be accomplished by choosing a low particle concentration of MRE. Alternatively, this adjustment can be completed by changing the geometry of the hemispherical shape’s height. The lower height, the lower the repulsive force is generated. The increment of the repulsive force of MRTTC is relatively simple since it is increased by just an increment of the magnetic field intensity. It is finally remarked here that the MR effect of MRF affects the repulsive force higher than the MR effect of MRE in the proposed MRTTC. This can be confirmed from SEM images shown in Figure 7. 

In fact, there are two approaches to get a wide range of the repulsive force of the proposed MRTTC. One way is to apply as high a magnetic field as possible. However, a high magnetic field may cause magnetic interference to adjacent magnetic-responsive sensors or/and any other medical devices in the robot-assisted surgery environment. This may happen before the magnetic saturation. Thus, in this work, the maximum current was limited by 0.4 A, corresponding to 0.2 T. The other way is to adjust the particle concentration of MRF and MRE. The higher repulsive force is achieved as, the higher particle volume fraction of MRF or/and MRE is used. The repulsive force can also be reduced by reducing the amount of the particles of MRF and MRE. However, in this case the spectrum of the repulsive force is limited due to the weak formation of the particle chain of MRF and weak particle interaction of MRE. Therefore, an appropriate choice of particle concentration of MRF and MRE with the allowable magnetic field is a key to achieve a wide spectrum of the repulsive force of the proposed MRTTC. This optimization will be undertaken as a subsequent study in the future. 

**Figure 14:**
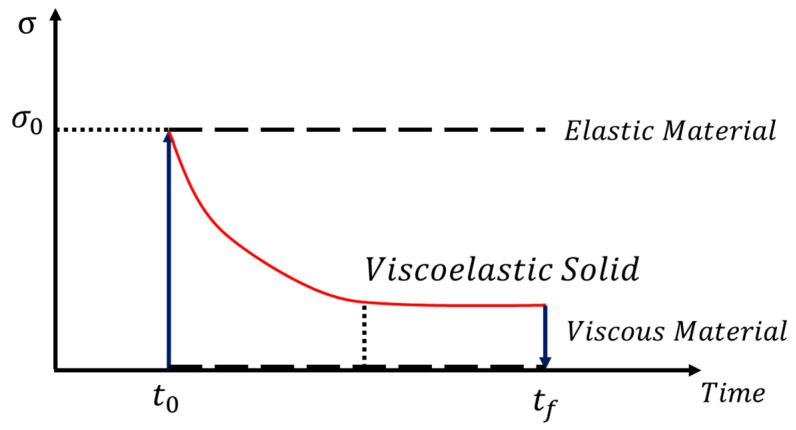
Stress relaxation behavior of the viscoelastic material.

It is seen from the field-dependent repulsive force measured with the proposed MRTTC that there exhibits stress relaxation behavior, which is a typical property of viscoelastic behavior. Thus, the field-dependent repulsive force can be modeled by the time-dependent stress curve, as shown in Figure 14. It is seen from the figure that the stress exhibits the peak value at the initial time $t_{0}$ and then falls exponentially to a lower equilibrium at $t_{f}$ which represents the viscous material property. Since the strain rate is zero during the stress relaxation, the stress can be expressed by:

(1) $$\sigma \left(t\right)=\sigma_{0}\;exp(-\frac{t}{\tau })$$

In the above, τ is the relaxation time when the stress falls to 1/*exp* (1e) of its initial value. It is noted that Equation (1) represents the stress relaxation in the absence of the magnetic field (or input current). By applying the different magnitudes of the magnetic field, the peak value at the initial time and the relaxation time are to be changed. In other words, $\sigma_{0}\left(H\right)$ and τ(H) are functions of the magnetic field intensity of *H*. These values are obtained from Equation (1) based on the experimental results of the field-dependent repulsive force shown in Figure 12. It should also be mentioned that instead of the unit stress, the units of force are used from the relation between the force and the stress because the applied area of the force is constant. Therefore, the field-dependent repulsive force can be expressed by:

(2) $$F{\left(H\right)}_{repulsive}=\{\sigma_{0}\left(H\right)exp\left(-\frac{t}{\tau \left(H\right)}\right)\}\times \left\{{Α}_{effective}\right\}$$

In the above, ${Α}_{effective}$ is the effective area of the compressive deformation. This area can be determined from the repulsive force dividing it by the corresponding stress.

**Figure 15:**
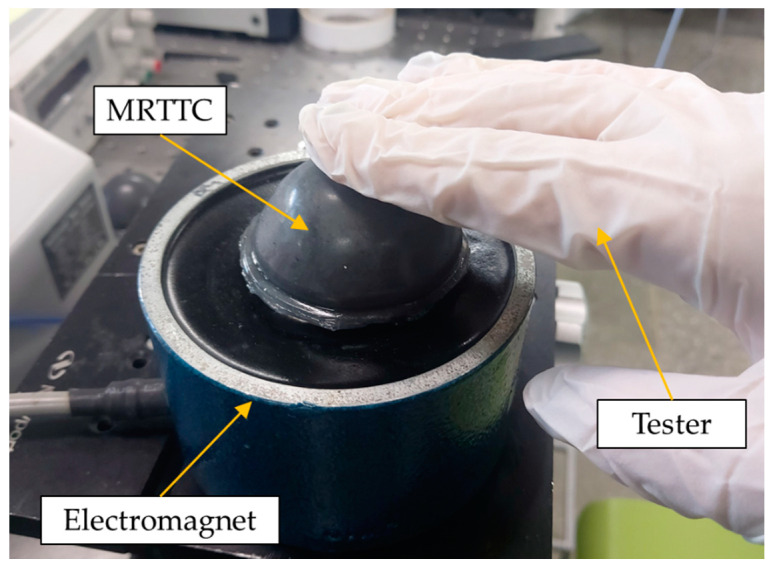
Apparatus for the psychophysical testing.

**Figure 16:**
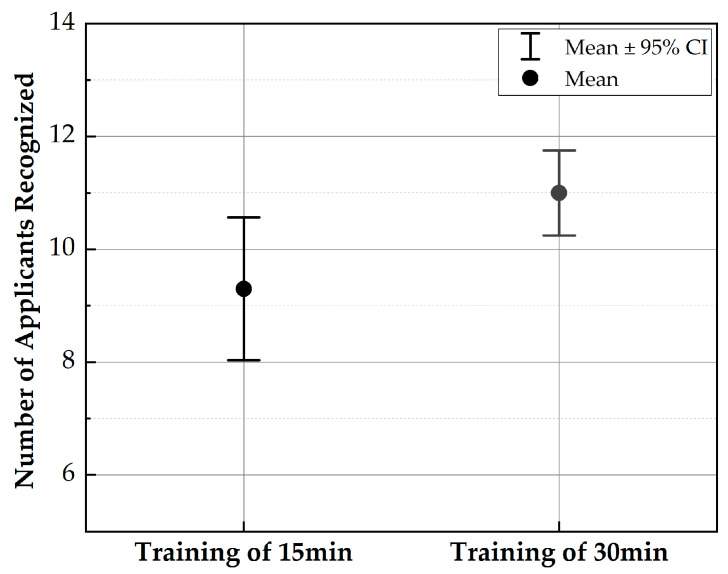
Result of the psychophysical testing with 20 volunteers.

In this work, a psychophysical test is also carried out to reflect the practical use of the proposed MRTTC. For the testing, the organs are divided into three corresponding to the skin (1.420~1.578 N), heart (1.578~1.894 N), and skeletal muscle (1.894~2.367). The value used in the experiment is, of course, based on the maximum value of the force range, and each corresponding input current is applied. As a preliminary test, 20 volunteers who are working on the design and control of medical robots participated in the investigation. Their ages are ranged from 20 to 30. The volunteers are given 15 and 30 min of training in the invisible state to discern the repulsive force corresponding to each organ. After training, they rested for 10 min and started the testing. A total of 12 tests are conducted by all volunteers where they wear latex gloves to touch MRTTC, as shown in Figure 15. The standard deviation is calculated by randomly implementing three organs’ repulsive force and recording the average value of the number of test results. The test is performed by dividing into A group (training of 15 min) and B group (training of 30 min) according to the training time. The longer the training time, the more sensitive and more accurate the perceived sensitivity becomes. Figure 16 shows the standard deviation of the two groups. From this result, it can be asserted that the volunteers can accurately identify different human organs with a short training time. This is one of the salient merits of the proposed MRTTC for application to RMIS. After conducting the test, the specific questions presented in Table 4 are surveyed. This question is recorded on a 5-point scale, and the scores for the responses are similarly averaged. The standard deviation is calculated using the average value. The questions at this stage are Question No. 1 is “Can the repulsive force of each sample be recognized?” and Question No. 2 is “Do you think that the repulsive force corresponding to each sample can be realized using the proposed tactile transfer device? (Use a 5-point scale)” to be. Question No. 3 is “How much do you think the accuracy of MRTTC? (Use a 5-point scale)” Based on these questions’ results, it has been confirmed that the proposed MRTTC can be effectively applicable to RMIS with a short training time for the sensation of human organs or tissues. However, it is remarked here that more volunteers should have participated in this test to have more reliable data for practical applicability. It is finally noted that one of the merits of the proposed tactile device MRTTC is to realize the different repulsive force with short training compared with other existing tactile devices such as piezoelectric-based ones. This is possible from the stress relaxation behavior representing the viscoelastic materials. Thus, we can undertake a blind test after training of 15–30 min. In this work, the volunteers who participated in this psychophysical test are graduate students who do not have any surgery experience. This directly indicates that the training time to identify a certain softness (or hardness) corresponding to a certain human organ can be shortened if the surgeons have the proposed MRTTC for the training. It is also remarked that one can easily predict the repulsive force by touching the proposed MRTTC subjected to a specific input current (or magnetic field) after training. This feature is also one of the merits of MRTTC as a tactile device for RMIS.

## 6. Conclusions

In this work, a new type of tactile device called MRTTC, which is applicable to robot-assisted minimally invasive surgery (RMIS), has been fabricated and its field-dependent repulsive force performance has been evaluated. The proposed MRTTC consists of two different magnetic-responsive materials: 1 mm thick MRE cover wrapping a polyurethane foam and a MRF core absorbed by the polyurethane foam was fabricated after analyzing the uniform distribution of the magnetic field. Subsequently, the field-dependent repulsive forces were measured at three different compression depths: 4, 6 and 8 mm. The maximum peak force at 0.4 A was identified by 0.959 N, 1.510 N and 2.783 N at 4, 6 and 8 mm, respectively. It should be noted here that the field-dependent repulsive produced from MRTTC is the same as the stress relaxation behavior of human organs even though both MRF and MRE exhibit nonlinear viscoelastic phenomena. Thus, Young’s moduli of human organs given in the literature are converted into a repulsive force using the finite element analysis method and compared with the measured ones. The similarity between the measurements and calculations directly indicates that the proposed MRTTC can be effectively applicable to RMIS where the surgeons need a tactile device to feel the same hardness of the organ to be operated on. The practical feasibility of the proposed MRTTC has been validated through a simple psychophysical test with 20 volunteers. It has been identified from this test that the standard deviation of 15 min training and 30 min training is evaluated by 1.68 and 1.00, respectively. 

As mentioned in the Introduction, the results achieved from this work are preliminary and show a possibility of the proposed MRTTC for application in an RMIS environment. Therefore, the following tasks should be completed to develop a successful MRTTC in the future: (1) The shape of MRTTC needs to be modified to adapt the repulsive form from any contact angle; (2) the optimization of the particle concentration of MRF and MRE to achieve a wider spectrum of the repulsive force without the magnetic saturation needs to be undertaken; (3) a thinner MRE layer to increase the touchable sensitivity due to the shallow compression depths needs to be fabricated and (4) the determination of an appropriate installation place of MRTTC in which the surgeon can feel the field-dependent repulsive force generated from the tactile device and the signal of the slave hand so that the surgeon can operate the master more accurately.

## Figures and Tables

**Table 1:** Young’s modulus of various tissues of humans [48,49,50].

Young’s Modulus of Human Tissue (Pa)	
Smooth Muscle	5000
Kidney	5000~10,000
Skin	9000
Skeleton Muscle	12,000
Heart	10,000~15,000

**Table 2:** Maximum repulsive force using the finite element method (calculated).

Compression Depth	4 mm	6 mm	8 mm
Kidney Low	0.395 N	0.592 N	0.789 N
Kidney High	0.790 N	1.184 N	1.578 N
Smooth Muscle	0.395 N	0.592 N	0.789 N
Skeleton Muscle	0.947 N	1.422 N	1.894 N
Skin	0.710 N	1.065 N	1.420 N
Heart Low	0.790 N	1.184 N	1.578 N
Heart High	1.184 N	1.775 N	2.367 N

**Table 3:** Peak force and relaxation force values for each compression depth.

Compression Depth		Input Current (A)	MRTTC (N)		Input Current (A)	MRTTC (N)
4 mm	Maximum Peak Force	0.00 A	0.412	Stress Relaxation	0.00 A	0.290
		0.13 A	0.608		0.08 A	0.431
		0.27 A	0.770		0.16 A	0.543
		0.40 A	0.959		0.24 A	0.626
6 mm	Maximum Peak Force	0.00 A	0.803	Stress Relaxation	0.00 A	0.554
		0.13 A	1.060		0.08 A	0.710
		0.27 A	1.306		0.16 A	0.840
		0.40 A	1.510		0.24 A	0.997
8 mm	Maximum Peak Force	0.00 A	1.120	Stress Relaxation	0.00 A	0.791
		0.13 A	1.159		0.08 A	1.042
		0.27 A	2.173		0.16 A	1.260
		0.40 A	2.783		0.24 A	1.567

**Table 4:** Psychophysical test survey for the proposed MRTTC.

Question	No. of Volunteers	Mean	Standard Deviation
Q.1 Can the repulsive force of each sample be recognized? (Use a 5-point scale)	20	4.25	0.72
Q.2. Do you think that the repulsive force corresponding to each sample can be realized using the proposed tactile transfer device? (Use a 5-point scale)	20	4.15	0.59
Q.3 How much do you think the accuracy of MRTTC? (Use a 5-point scale)	20	4.25	0.55

## Data Availability

Not Applicable.
